# How to do things with metaphors: engineering life as hodgepodge

**DOI:** 10.1186/s40504-018-0084-z

**Published:** 2018-09-17

**Authors:** Matthew Kearnes, Declan Kuch, Angus Johnston

**Affiliations:** 1 ARC Centre of Excellence in Convergent Bio-Nano Science & Technology (CBNS), School of Humanities and Languages, University of New South Wales, Sydney, Australia; 2 ARC Centre of Excellence in Convergent Bio-Nano Science & Technology (CBNS), Monash Institute of Pharmaceutical Sciences, Monash University, Melbourne, Australia

## Abstract

This paper presents a collaboration between social scientists and a chemist exploring the promises for new therapy development at the intersection between synthetic biology and nanotechnology. Drawing from ethnographic studies of laboratories and a recorded discussion between the three authors, we interrogate the metaphors that underpin what Mackenzie (Futures 48:5-12 2013) has identified as a recursive relationship in the iconography of the life sciences and its infrastructure. Focusing specifically on the use of gene editing techniques in synthetic biology and bio-nanotechnology, we focus our analysis on the key metaphors of ‘evolutionary life as hodge-podge’ within which ‘cutting’ of DNA and the ‘sticking’ and ‘binding’ of engineered particles to proteins can be performed by researchers in laboratory settings. Taken together, we argue that these metaphors are consequential for understanding metaphors of life-as-machine and the prevalence of notions of ‘engineering life’. Exploring the ways in which notions of cutting, targeting and life as an evolutionary hodgepodge prefigure a more contingent notion of engineering and synthesis we close by considering the interpretive implications for ethnomethodological approaches to contemporary life science research.

## Introduction

In their history of genetic research – and the more recent development of genomic and postgenomic paradigms across the contemporary life sciences – Barnes and Dupré (2008) – contend that “chromosomes and their DNA need to be understood as material things all the time, even when they are transferring information” (p. 66). This insistence that DNA be understood materially is set in the context of the stock of informational metaphors deployed to represent the contemporary life sciences – that speak of a capacity to re-write, and indeed to cut and paste from, the ‘Book of Life’.^1^ Metaphors of writing, re-writing, and editing are indicative of the ways in which the contemporary biosciences are characterised by intense and overlapping forms of (inter)textuality (Landecker 2007). However, in pursuing a more materialist reading of the contemporary life sciences Barnes and Dupré (2008) continue by suggesting that while “informatic metaphors may usefully be applied to” DNA transcription “to understand… how rapidly and accurately transcription proceeds it is necessary to remember that it is a process involving specific materials” (p. 66).

This insistence on the materiality of DNA can be understood as an attempt to re-tell the history of genetic research in the context of what is increasingly presented as a ‘postgenomic’ turn in contemporary bioscience research (Reardon 2017). This ‘turn’ can be further characterised by ways in which foundationalist and context-independent accounts of the gene are being challenged by what Meloni (2013) refers to as the “postgenomic appreciation of a bi-directional interaction between ‘the biological’ and the ‘environmental’” (p. 742). As bioscientific research is increasingly characterised by a fluid traffic between a range of ‘omes’ – the ‘epigenome’, ‘proteome’ and the ‘microbiome’, for example – and across the organic/inorganic interface, the life sciences have acquired both a temporality and a geography (Stallins et al. 2018, Lappé and Landecker 2015, Lock 2015). In this sense, one of the central ironies of the contemporary biosciences is, therefore, the ways in which a postgenomic reading of what Lock (2001) terms ‘local biologies’ – an insistence on the “recognition of the embodiment of an historicised biology” (p. 73. See also Meloni 2014) – sits alongside the promise of spatially precise manipulation – even editing – of DNA. In an era where the life sciences have appeared to “stand poised to serve both state ambition and private desire” (Jasanoff 2005, 36), the circulation of images and videos that depict the deployment of gene editing techniques in manipulating DNA in real time, cutting DNA in two and ‘snipping’ strands of DNA^2^ prefigures promissory accounts of tailored genetic therapies, synthetic biology and precision medicine.

How then might a materially sensitive understanding of the biosciences be taken up in readings of the metaphorical and analogical terrain of fields such as synthetic biology and bio-nanotechnology? In this paper, we are interested in how metaphors of precise gene editing in research at the interface between synthetic biology and bio-nanotechnology, and associated particularly with the recent development and diffusion of CRISPR-Cas9 techniques, are taken up and deployed in situated laboratory contexts. We argue that the metaphorical and textual terrain that maps the interface between the life and material sciences, that Rheinberger (2003) terms the “scrips and scribbles of the laboratory”, provide both an interpretive register in the formulation of epistemic objects whilst at the same time constituting a site for normative enquiry and political contestation (McLeod and Nerlich 2017). Rather than the truth (or otherwise) of metaphorical representations of synthetic biology and gene editing (Nelson et al., 2015), we argue that metaphorical formulations – that speak of the capabilities and capacities afforded by gene editing – offer a ‘navigational resource’ in charting the cultural meanings of bioscientific research in a cultural context increasingly defined by both the proliferation of promissory narratives and the emergence of a more ambivalent and reflexive attitude toward promises of technological breakthroughs and progress (Kearnes and Wynne 2007, Kerr and Cunningham-Burley 2000, Pickersgill 2013).

By emphasising the pragmatic and performative deployment of metaphors in the uptake and diffusion of CRISPR-Cas9 techniques, across fields such as synthetic biology and bio-nanotechnology, we are drawing on Balmer et al. (2016a) assertion of the importance of attending to synthetic biology *in situ*. Our analysis is based on two claims. The first is that the development of gene editing techniques, such as CRISPR-Cas9, increasingly form a underpinning technical capability of research in fields such as synthetic biology. For example, a recent review of the engineering of synthetic gene regulation circuits suggested that CRISPR had become a “notable addition to the *circuit engineering toolkit* … which has been used as tool to recruit transcriptional machinery to specific genomic loci and to construct multi-node circuitry” (Bashor and Collins 2018, 410, emphasis added). Implicit in the vocabulary of synthesis and the imagery evoked by synthetic biology are capabilities for precise manipulation of genetic material, through the techniques of gene editing. Our second claim builds on the notion that the diffusion of CRISPR across the life sciences blurs the distinction between synthetic biology and other fields of research. For this reason, rather than assuming that fields such as synthetic biology are defined by a prima facie novelty, characterised by unique technical apparatuses, epistemic cultures and technological and societal outcomes, Balmer et al. argue for an account of the emergence of synthetic biology through situated material and epistemic enactments.

Building on this approach we argue that a notion of the materially situated deployment of metaphors, that are often promissory in nature and intent, implies analytical consequences for their interpretation. Developing his account of promising in the light of performative analyses of speech acts developed by J. L Austin (1962), Mike Fortun (2008) argues for an account of promising that extends beyond human agents, and encompasses material agents, in situated contexts. He suggests that “promising is always an event involving, and evolving from, an amalgam of language and matter” (p.104). In his earlier work, Fortun (2005) takes this Austinian reading of the performativity of promising further, noting that, “the rhetoric of the promise is everywhere in genomics, and it’s all too easy and all too tempting to dismiss or overlook the real paradoxes of promising, and either take such statements at face value, or dismiss them as ‘mere hype’” (p. 158). He instead argues that, “promising cannot be reduced to either empty hype, or to formal contract, but occupies the uncertain, difficult space in between” (p. 158). In place of what he terms “a conservative, preservationist bioethics” that might be “necessary in our encounters with the excesses of biotechnoscience” – that sees metaphorical constructions as an obstacle to a critical interpretation of the social and ethical dimensions of novel fields – Fortun argues that it is “even more necessary that we supplement [such a bioethics] with other ethical strategies or styles that would gamble on and, with luck, capitalize on the excesses of promising” (p. 165). The notion of excess that Fortun marshals here is not simply rhetorical – and yet where this promise is increasingly scrutinised – promissory scientific metaphors are deployed in performative enactments that order the world in ways that make the realisation of these promises (at least partially) possible (Mackenzie 2013).

In this paper, we extend Fortun’s notion of the excess of promising by exploring the ways in which situated laboratory practices are entailed in organising the world into metaphorical constructs in ways that are materially and socially excessive. This paper was written in the context of an ongoing collaboration between the authors.^3^ Recent work in science and technology studies, has explored the vicissitudes of collaborative modes of engagement between the natural, physical and social science. This work has documented both the possibilities for post-ELSI interdisciplinary collaboration (Balmer and Bulpin 2013, Balmer et al., 2016, Balmer et al. 2015), where the laboratory becomes a site for collaborative ethnography and engagement (Gjefsen and Fisher 2014), and has cautioned “against integration as a novel mode of governance” (Viseu 2015, 642). In this paper we explore the methodological possibilities for the collective explication and interpretation of metaphors as a promising mode of collaboration in the context a new relationship between social and natural science that seeks to avoid the comforting assurances of “suspicion, antagonism, opposition, conflict [and] distrust” (Fortun 2005, 160).

In the following sections we draw on ongoing ethnographic engagement with researchers working in synthetic biology and bionanotechnology, in order to develop a collaborative mode of writing and interpretation.^4^ In the following sections we explore the metaphors cutting and editing genetic material, together with the proto-ontological metaphor that presents life as an evolutionary hodgepodge. In closing we suggest that this hodgepodge metaphor evokes a contingent notion of synthesis and design and thereby represent an alternate conception for what many have identified as a postgenomic turn toward the engineering of biologic materials.

### Cuts, edits and knock-outs

For a field of research that trades – in both its scholarly and popular representations – on notions of its timeliness and ‘breakthrough’ potential, synthetic biology seems curiously consumed with narrating its own history and maturity.^5^ Early accounts of the field – in a series of expert reports, manifestos and popular editorials largely were consumed with ‘announcements’ of the emergence of a new field – declaring the development of “new engineering rules for an emerging discipline” (Andrianantoandro et al. 2006), a “new frontier” in biomedical research (Doudna and Charpentier 2014) and the discovery of “new worlds” (de Lorenzo and Danchin 2008) complete with commercial and technological roadmaps (Lux Research 2009, UK Synthetic Biology Roadmap Coordination Group 2012) and prominent synthetic biology researchers.^6^ At the same time, a second narrative emerged that countered notions of novelty with accounts of the maturity of synthetic biology – that synthetic biology had come of age (Khalil and Collins 2010) – and of the prospects for a ‘second wave’ of synthetic biology research (Purnick and Weiss 2009).

While this segmentation of synthetic biology research into successive waves is largely arbitrary, it is notable that these accounts of synthetic biology appear to share a ‘biologism’ (Meloni 2013); and are told from the perspective of the kind of biological foundationalism that has characterised much of the cultural histories of the life sciences (Barnes and Dupré 2008). In contrast, in her attempt to chart the emergence of synthetic biology Bensaude Vincent (2013) charts the parallel histories of the notions of synthesis in both biology and chemistry to probe how alternative research trajectories – based in the histories of bioengineering and biochemistry – were articulated through the deployment of a distinct repertoire of different analogies and metaphors. Counterpoising the computational metaphors of “standardisation, modularisation, interoperability, transparency and reliability” (p. 124) – most commonly associated with bioengineering – with those of bio-inspired chemical synthesis, Bensaude Vincent (2013)^7^ demonstrates that these two parallel histories entail a divergent set of metaphorical constructs and are entangled with differing social and political stakes. Arguing that due to their different conceptions of “knowing and making” Bensaude Vincent suggests that these “two models of synthesis do not engage the designer’s responsibility in the same manner” (p. 127). As a consequence, “whereas the algorithmic approach to synthesis inspired by engineering requires a blueprint of the process to make it predictable, the chemical approach always allows surprise, hazards and opportunities to occur” (p. 127).

The two parallel notions of synthesis that Bensaude Vincent identifies – the computational and the chemical – remain operative, and largely unresolved, in fields such as synthetic biology and bio-nanotechnology.^8^ At the same time, one index of the metaphorical bricolage that characterises research across these fields is ways in which the techniques of gene editing, such as CRISPR-Cas9,^9^ and DNA assembly are increasingly regarded as infrastructural capabilities across contemporary biomedical research. As we suggest above, gene editing is metaphorically taken up as part of the ‘engineering toolkit’ of contemporary synthetic biology and bio-nanotechnology. Rather than constituting unique fields of enquiry, the conceptions of synthesis that Bensaude Vincent identifies – complete with biologically and chemically derived metaphors – are taken up in the situated work of coordinating the use of gene editing techniques in experimental settings. As we met together to plan the writing of this paper, Angus explained the ways in which CRISPR-Cas9 techniques had been implemented in his work exploring the dynamic interactions between nanomaterials and biological systems. Our conversation began with a discussion of research in bionanotechnology, specifically focused on the development of targeted drug delivery systems. In his written work Angus has outlined the ways in which his work is situated in long-term research agendas on targeted drug delivery. “Fundamental to effective drug delivery”, Johnston (2017) writes, is “transporting drugs to the specific subcellular locations where they are therapeutically active” (p. 4). In the context of the development of novel drug delivery systems, he notes that although “there has been an explosion of interest in nanoparticle systems … the therapeutic outcomes have largely been hit or miss”. For Johnston (2017) three key parameters are critical to the ongoing development of nanoparticle research: “(1) Does the nanoparticle enter the cell? (2) Where do the nanoparticle and drug go inside the cell, and how do they get there? (3) What is the local environment that the nanoparticle is exposed to, so a release mechanism can be engineered to deliver the drug when it reaches the required location” (p. 4).

In research in targeted drug delivery the turn toward gene editing techniques is conceived as a way of overcoming the barriers that living cells present to engineering materials. In our conversation, as we turned to these issues Angus remarked modestly, “we use CRISPR-Cas9 in a very basic way, just for cells lines that we want to study, if we want to knock out a particular gene”. The attraction of CRISPR-Cas9 and gene editing technologies is in overcoming biological barriers that limit the uptake of engineered nanoparticles. Angus then commented that in his ongoing research, “one of the key things is being able to edit the genes of the organism to be able to either get it make a new protein or change the proteins in some way”. And while he noted that “CRISPR-Cas9 wouldn’t be your first choice” for manufacturing proteins for injection into mammalian or eukaryotic cells, “the huge advance of CRISPR-Cas9 is that rather than just sticking DNA in somewhat randomly CRISPR-Cas9 enables you to put it in a specific spot so you can put a new protein into the genome in the exact spot that you want”.

Critical to the language of precision, placement and intentionality in representations of the potential of CRISPR-Cas9 and gene editing are metaphors of cutting, editing and knocking-out elements of DNA. The notion of precise gene editing, and the language of cutting DNA, is also central to the realisation of “the promise of biology as technology” (Mackenzie 2013, 6), embodied in biological systems that might be harnessed as allies, rather than barriers, in the development of targeted and precision medicine. In our conversation, Angus clarified the ways in which gene editing techniques had entered his research:

The Holy Grail of CRISPR is that if people have genetic disorders you can go in and say okay we know where that DNA code is incorrect. We can use CRISPR but to edit your own genome and only correct the spot that needs correcting. We don’t have to cut out a large chunk of DNA, we don’t have to stick a great big new piece of DNA in, you will have your old bit of DNA that wasn’t working. You design the CRISPR protocol that you’re trying to do to edit down to a single nucleic acid mutation in the entire genome. It will recognise it and fix it. You can chop something out. You can put something in or you can edit. That’s the thing that it does, it’s an editing tool but an editing tool with really powerful accuracy.

The development of gene editing techniques, are deeply interwoven with the nest of linguistic and informational metaphors that have characterised the parallel histories of molecular biology and organic chemistry. Writing on synthetic biology, McLeod and Nerlich (2017) argue that the field “is grounded in three ‘big’ metaphors,” namely of organisms as books, organisms as machines, and organisms as computers. In this sense, in the metaphorical construction of synthetic biology, linguistic metaphors speak to a conception of both the organism – and of DNA specifically – as readable, malleable and plastic (Landecker 2010) and the industrial potential of a machine-like understanding of biological processes. McLeod and Nerlich (2017) situate this metaphor in the context of three industrial revolutions: “the printing revolution initiated by Gutenberg in the 1400s, the industrial revolution grounded in new types of engines, engineering and machines that started in the 1800s and bringing with it standardised parts, mass production and assembly lines, and the computer or information revolution that began in the mid-1900s” (p. 8). In this sense, linguistic metaphors drawn from everyday computing – such as the ‘find and replace’ function in Word, and editing in Photoshop – capture the virtualism of manipulation through digital interfaces that are increasingly entangled with algorithmic systems.

These linguistic and informational metaphors – that speak of cutting and pasting DNA code and of the customizability of genes – are an index of the traffic between biology, chemistry and the information sciences first instantiated in the confluence between molecular biology and cybernetics (Keller 1995, Kay 2000). Notions of cutting and replacing DNA code seem to traverse two alternative accounts of rendering the biological machine-like. Metaphors of biological machinery have constituted some of the most resilient constructs across the life- and bio-sciences (Keller 2002). Thus, a characteristic feature of representations of synthetic biology is a confusion between ontological claims concerning the biological (the biological *is a* machine) and more pragmatic notions of design and modularity (the biological can be *made* machine-like). This in turn has propelled two alternative notions of design in synthetic biology – where accounts of biological machines produced through the creation of modular biological parts (Frow and Calvert 2013) are set against biomimetic notions of harnessing the a priori machine-like qualities of biological systems (Mackenzie 2010).

Mackenzie (2013) develops the notion of ‘infrastructural icons’ from Bowker’s (1995) concept of ‘infrastructural inversion’ to depict the duality of this elision of machine and life –the ways in which “iconic forms become more infrastructural as they develop”, while at the same time suggesting that “synthetic biologists iconify infrastructures” (p. 10). For Mackenzie, the process by which iconic forms become infrastructures and vice versa *–* whereby CRISPR appears as both a revolutionary breakthrough and a pragmatic technique – is located in the gap between the social, economic and political promises of a gene editing and their realisation, rather than simply being an index of the technical maturity of a field. The gap here is a promissory horizon wherein counter-narratives such as off-target effects (Kleinstiver et al. 2016), concerns about allergies and cancer (Schaefer et al. 2017a) intrude repeatedly. In this context, what is striking about metaphors of cutting, pasting and knocking-out DNA, is that they constitute a resource for both linking and navigating the relationship between proto-ontological claims concerning the nature of biological substance, grand accounts of the bio-economic potential of fields such as synthetic biology and the practical work of organising and ordering the epistemic materials of biomedical research. The notion of cutting and replacing DNA appears simultaneously as an ontic claim about the nature of DNA – where DNA is presented as *cut-able* – and as an infrastructural claim where cutting is presented as a technique and capacity that, while revolutionary, is quickly becoming standardised and mundane.

### Life as hodgepodge

Critical to the deployment of the metaphors of cutting and editing is the notion that cuts are made precisely, at specific genetic locations. For example, in their study of the representation of CRISPR technologies in the US popular media O’Keefe et al. (2015) find that in addition to metaphors of ‘editing’ notions of ‘targeting’, and that cutting DNA would be guided with precision accuracy, dominate the discursive repertoire in the public representation of CRISPR. While both of these metaphors are torqued toward a series of promissory narratives, that CRISPR technologies will enable an ability to alter DNA at specific sites with both speed and precision, O’Keefe, et al. (2015) are concerned that both of these metaphors are “misleading”. They suggest that both metaphors imply “a pattern of reduced complexity and exaggerated control of outcomes that has troubling implications” (p. 5) and they note that in particular the metaphor of targeting functions to “warn of the dangers of unintended cuts” (p. 7). In this sense, the paired metaphors of editing and targeting “assume successful aim” (p. 8) and are strategically deployed to “address the problems that can arise when targeting fails” (p. 8), particularly the acknowledged problem of ‘off-target effects’ in the use of CRISPR techniques.^10^ The infrastructural inversion that Mackenzie (2013) identifies in synthetic biology research is, in the case of CRISPR techniques, situated in the context of a live public debate about the possibility for ‘less-than-targeted’ outcomes of gene editing,^11^ and more recent suggestions that “editing cells’ genomes with CRISPR-Cas9 might increase the risk that the altered cells, intended to treat disease, will trigger cancer” (Begley 2018, no pagination).^12^

In this context, metaphors of cutting and targeting appear as a strategic resource for presenting gene editing simultaneously as a technology and an outcome; as depictions of gene edit*ing*, as an active verb, and as precisely edit*ed* genes.  Nelson et al., (2015) suggests that this conflation between what CRISPR *is* and what it *does* emphasises “the agency of scientists” through an articulation of an “implied locus of control” in the ‘editor’ (p. 61). Problematising this unitary notion of control O’Keefe et al. (2015) argue, that “we need metaphors for CRISPR that indicate the technology’s uncertainties and unknowns” and that “ecological metaphors could reflect the broad-ranging effects of modifying genomes and the fact that CRISPR is being used in ways that affect not only organisms but ecosystems themselves” (p. 8).

This move toward an ecological sensibility is striking, given the ways in which metaphors of gene editing, cutting and targeting are deployed in laboratory contexts in ways that evoke an ecological understanding of life and evolution. A notable example of such an ecological sensibility, invoked by metaphors encountered during our ethnographic interactions, is the way cutting and editing metaphors are situated in accounts of the messy nature of life itself. It is common, for example, for researchers to draw on analogies of evolution as a jumble of things thrown together and constantly requiring repair and adaption, rather than a well-oiled, finely engineered machine. This analogy prefigures a seemingly fragile and contingent notion of engineering, in contrast to the more unitary images of technological precision and control that characterise the metaphorical terrain of fields such as synthetic biology and bio-nanotechnology. Boudry and Pigliucci (2013) summarise this more provisional notion of design as a movement from “human-made machines” to “the level of molecular biology and genetics”. At this scale “living organisms are far more messy and less transparent than human-made machines. Notoriously, evolution is an opportunistic tinkerer, blindly stumbling on ‘designs’ that no sensible engineer would come up with” (p. 660). Life figured as an evolutionary *hodgepodge*, that is held together precariously and provisionally at the molecular level, emerges as an ontological metaphor that prefigures a notion of engineering as tinkering. In our conversation, Angus evoked this more careful, and contingent, understanding of gene editing:

**Angus**: …for the protein engineering, we know we need resistance to an antibiotic because that’s the way we select for the proteins that we have. So we’ve got that part. We put in meat and we have a part in putting unnatural amino acid in, we have a part for the new protein we make, we put five or six parts together and it makes our machine. So [the engineered organism] is modular to an extent but it’s modular to a baseline of a machine that’s ticking away, that most of what it’s doing is something completely irrelevant for what we’re trying to do. But we don’t really care because we get enough out of it at the end to do what we need to.

**Matt**: It’s almost like biohacking.

**Angus**: Yes it is. Oh yeah it is, it is, and yet you are hacking into an organism. It’s … like we are hacking, or more like a parasite, it’s trying to do what it normally wants to do and we just put something in. Basically if it realised what we were trying to do it would try and spit it out and not do it but we trick it to think ‘Well it’s doing what we want to do’ and it’s just trying to continue on to do what he wants to do.

**Declan**: Did you ever see that show Bush Mechanics?^13^

**Angus**: Yes yeah – that’s a good analogy of how evolution has worked. It’s not a finely oiled machine. It is a hodgepodge of things that really don’t work that well, but they work well enough to meet certain ends. There are certain enzymes which you need to have half a dozen of to do a fairly basic process and you think, ‘well why don’t we just have an enzyme that does that thing in the first place?’ But it’s because the way it evolved, it was never a goal to make that particular substrate.

The metaphor of nature as hodgepodge, that Angus evokes here, reinforces a ‘post-organismic’ view of life, whereby “the biological features researchers fasten on are determined by their own experimental tactics, which they then identify with the thing itself… that is, they identify their manufacturing methods as inherent in biology prior to their own interventions” (Roosth 2013, 167). In this sense the metaphor of life-as-hodgepodge appears as both a proto-ontological claim and a pragmatic rendering of the material practice of gene editing research. O’Malley (2011) captures something of this metaphor by developing the terminology of kludging, “a colloquial term for a workaround solution that is klumsy, lame, ugly, dumb, but good enough” (p. 409). When life is figured as an evolutionary hodgepodge, notions of design and engineering – and of harnessing the evolutionary potential of biological systems – appear as a form of kludging that “emphasises functional achievement, rather than the way in which that function is achieved” (p. 409). Angus’ reference to the popular TV show Bush Mechanics, might therefore be read as a evoking a notion of kludging in an Australian vernacular.

This understanding of the dynamism of evolutionary processes complicates what some have seen as reductionist project characterised by the application of engineering ‘mind-set’ in a biological context. For Rabinow and Bennett (2012), this mind-set is a defining feature of synthetic biology. They argue that “post-genomics has seen the intensification of an engineering disposition in biology: understanding through making and remaking” and that “the challenge for synthetic biologists is to take biology beyond the guild-like restrictions of artisanal *savior faire* and to make it into a fullfledged engineering discipline, with all this entails in terms of standardization, modularization, and regularization” (pp. 16–17). However, in place of a rather unitary notion of engineering, the metaphors of engineering and arrangement marshalled by Angus suggest a more pragmatic ethos, defined by tinkering, hacking and making rather than authorial design. Building on Bensaude Vincent’s (2013) account of the parallel notions of synthesis that characterise fields such as synthetic biology, in the deployment of gene editing techniques notions of ‘design as blueprint’ and overlain by an alternative metaphor of ‘design as emergent’, contributing to an altogether less than heroic notion of engineering.

At the same time, this more pragmatic and contingent notion of biological manipulation complicates images of factory-like biological machinery that are often associated with synthetic biology; embodied in metaphors of interchangeable biological parts assembled into chassis. Our discussions turned to how, in ‘hacking an organism’, it is possible to distinguish between ‘good’ and ‘bad’ binding of proteins. Practically, protein interactions mean, “bringing the thing that you want to have stick come to the surface, then you change that surface, then look to see whether it still sticks.” The problem with this approach is that binding may be specific or non-specific to the surface. When the surface is switched, and you have a cell that you would not want to detect, it might stick very well but still be a ‘bad’, non-specific binding. There are two methods easily available to understand these interactions: Firstly, fluorescent tagging allows Angus to track where threshold numbers of molecules accumulate within a cell because of the availability of light detectable through his microscopes. A second approach is to add newly engineered materials to well characterised materials:

Angus: “we deliberately block the binding so we know we have the receptors on the surface of the cell and then we flood it with something that we know sticks to that receptor and then we bring in our new engineered material and we see if it can still bind. So they’re the two ways that we see if it’s specific, meaning is that only recognizing the receptor that we want? We can’t actually see individually which receptors it sticking to. It doesn’t stick to the cell when the receptors are not there. It does stick to the cell when the receptor is there but that’s still not enough to be absolutely sure because sometimes by putting your receptor on the surface it changes something else in the cell. And so that it’s still not the receptor you’re interested in. But then if you go and block that receptor site, cover it up with another protein or something and then you stop binding, that’s when you can be quite sure that we’re getting that interaction with the specific protein that we designed it to be.”

The metaphors of sticking and binding at the interface between synthesized biological systems and engineered materials complement the hodgepodge metaphor. Viewed historically these metaphors of surface binding evoke the contemporary unfolding of receptor theory that has shaped bio-medical throughout the twentieth century. Most famously initiated in Paul Ehrlich’s side-chain-theory, specifically in his work on chemotherapy and infectious diseases such syphilis (Maehle 2009), the notion of optimising the biological reception of engineered materials constitutes a fundamental element of the notion of a ‘magic bullet’, the quest to discover a “*therapia sterilisans magna*, in other words, a treatment which could, in a single dose, destroy all microorganisms in the infected organism … without affecting the host’s cells” (Bosch and Rosich 2008, 175). Notions of sticking and binding function in two ways – presenting CRISPR itself as a magic bullet, whilst at the same time presenting a more infrastructural vision, of CRISPR as simply a tool to enable the creation of magic bullets. However, in the context of ongoing debates about the possibility of off-target effects of CRISPR – that problematise the ballistic metaphors of the magic bullet – the ‘life-as-hodgepodge’ metaphor, and its implied notion of engineering and kludging seeks to resolve this problem through the socio-technical infrastructures of visualisation and standardisation.

## Discussion and conclusion

Analyses of metaphors and analogies have constituted a critical method in the development of interpretive and ethnomethodological approaches in the field of science and technology studies. In contrast to the notion that the use of metaphors is simply decorative or strategic, this body of research has documented the ways in which analogical narratives are wrapped up in the process of constituting epistemic objects (Rheinberger 1997), defining new fields of research (Bensaude-Vincent and Loeve 2014, Molyneux-Hodgson and Meyer 2009) and demarcating notions of public value, responsibility and accountability (McLeod and Nerlich 2017). How then might we interpret the metaphorical terrain that underpins fields such as synthetic biology and gene editing? We have argued that this terrain draws liberally from the information sciences and analogies with popular computing – evoking ‘cut and paste’ orientation toward gene editing – whilst at the same time presenting techniques such as gene editing and CRISPR as ‘too good to the true’ (Scott 2018). In as much as fields such as synthetic biology and gene editing evoke metaphors of reading and writing the biological, drawn from a ready stock of analogical resources, we also see that this deployment is inventive and constructive, woven into the interpretive task of making biological writing culturally, socially and politically tractable.

The metaphorical terrain that underpins fields such as synthetic biology, and the techniques of gene editing, are therefore likely to continue to be sites for political deliberation and contestation. Jasanoff, Hurlbut, and Saha (2015) suggest that “the emergence of a far-reaching technology like CRISPR is a time when society takes stock of alternative imaginable futures and decides which ones are worth pursuing and which ones should be regulated, or even prevented” (no pagination). It is for this reason that we have argued that approaching metaphors, often characterised by narratives of opportunity and threat, ethnomethodologically requires attending to the situated contexts in which they are deployed. We have argued that notions of cutting and editing DNA, sticking and binding, and ‘life-as-hodgepodge’ are both consequential for the practices of contemporary bioscientific research whilst also operating as ‘navigational resources’ that enable researchers to chart a course through the contested cultural meanings of bioscientific research. In this sense, we have suggested that the representational adequacy of metaphors, should be contextualised in the pragmatically arranged and multiple practices of the laboratory, rather than against abstract or foundational criteria. In this instance, we have highlighted the flexibility with which techniques like CRISPR – and the metaphors that condition and shape its deployment – are enrolled in bioscientific work and laboratory contexts.

Three key points follow: Firstly, emphasising the flexibility of laboratory practice has important implications for the demarcations of synthetic biology and its relationship to life. Our analysis suggests that strong demarcations between the inside and outside of ‘synthetic biology’ as a field should be avoided. Strong demarcations may be useful for shoring up disciplines – in the definitional work necessary to secure political capital (Kearnes 2013) – but risk occluding the fluid traffic of techniques into and out of the laboratory. A second, and related, issue concerns the stakes of arguments concerning the materiality of DNA and gene editing. Metaphors of cutting and binding are often adjudicated as concepts against which distances between words and the world can be measured. This correspondence theory of truth (Latour 1999) risks obscuring the contingency and situatedness of how gene editing techniques are assembled in laboratories. In other words, the metaphor of ‘life-as-hodgpodge’ operates in service of the pragmatic assembly of materials in the laboratory. Thus, thirdly, we contend that while much has been made of the ways in which the panoply of post-genomic research agendas – such as synthetic biology and gene editing – represent the “intensification of an engineering disposition in biology”, where understanding is forged through “making and remaking” and “living systems, and their components, are being redesigned and refashioned” (Rabinow and Bennett 2008, 7) these developments must be contextualised by a reading of the materiality of the biological. The stakes implicit in the synthesis of new biological artefacts is not recreation of ‘life’ as a grandiose, quasi-theological concept, but rather a material – and often contingent – assembly of compounds that are engineered to fulfil specific and contestable criteria. This materialist notion of biological writing – captured by the metaphors of editing and targeting – suggests that tinkering, hacking and making might be more adequate metaphors for contemporary bioscientific research.

None of these arguments are intended to diminish the novelty and significance of gene editing techniques for scientific practice, but rather to contest the stakes of its deployment. If CRISPR is to be the vanguard of the second-wave of synthetic biology, metaphors of its interface with biological materials are highly consequential. Industrial analogies between synthetic biology and construction materials (bricks, screws etc.) should be taken advisedly. Technical coordination *about* life – even in the pragmatic senses we have discussed in this paper – requires humility about the prospects for its (re)engineering through such actions as cutting and editing. The more contextual understandings of bio-medical intervention through social determinants of health, epigenetics, and public health genomics – and the insistence on a situated and embodied sense of local biologies (Lock 2001) – gestured to at the beginning of this paper may offer a valuable starting point in instilling this humility. For this reason, the argument we have sought to advance here is that attending to the metaphors of life as a hodgepodge – where the creation and manipulation of biological materials appear as ‘kludged’ outcomes (O’Malley 2011) – offers an alternative vantage point for approaching questions concerning responsibility and social outcomes. What we have attempted to open up in this collaborative paper is a modality of thinking responsibility that takes as its inspiration Fortun’s (2005) notion of an ‘ethics of promising’. This mode of collaboration necessarily entails imaginative work. Working with similar themes, Stilgoe (2015) argues that scholars working on the social meanings of science and technology are “at their most useful when they are focussing not on science as knowledge, but as experiment, with the experiment in question being as much social as technical” (p. 51). Working in a collaborative vein, in this paper we have sought to advance a similar orientation: to see in metaphorical accounts resources that are creative and also pragmatically useful. The interpretation of synthetic biology metaphors is therefore as much inventive as it is documentary. As such, a mode of collaborative writing of biotechnological futures capable of sustaining a robust logic of responsible innovation might begin from the insistence that this inventive metaphorical work might be torqued to divergent ends.

## Footnotes

1 As we will explore below the notion of ‘rewriting the book of life’ is particularly associated with gene editing techniques such as CRISPR-Cas9. Writing for the Boston Consulting Group Boglioli and Richard (2015) capture something of the breathless hubris of this vision: *With this easier access to DNA sequences, today we are on the verge of a third revolution that will deeply impact our lives, to the extent that computers have changed society: we are entering the era of “gene editing”, following the era of “gene reading”. Gene editing is the rational and precise modification of DNA sequences program in living cells and organisms. Why edit genes? For everything: from designing pathogen-resistant crops or therapeutic correction of defective genes responsible for diseases to rewriting the program of organisms to produce new sophisticated biologicals*. (pg. 1, emphasis in original)

2 For example, a video that accompanied high-speed atomic force microscopy images that depicted gene editing in real time, produced by Shibata et al. (2017), circulated widely in the popular press and on social media (Zhang 2017). At the time of writing, the original tweet that publicised this work had been re-tweeted 3616 times (see: https://twitter.com/hnisimasu/status/928933260159197184).

3 This collaboration is enabled by the Australian Research Council (ARC) Centre of Excellence in Convergent Bio-Nano Science and Technology (CBNS). In this paper we draw on a period of ongoing ethnographic research between 2015 and 2018, conducted in the context of this research centre.

4 Three authors recorded a 120 min dialogue, focused specifically on the take-up of CRISPR-Cas9 techniques in bio-nanotechnology and synthetic biology research, which forms the basis of the discussion in the following sections. Taking our cues from both traditional social science methods of interview and ethnography, and recent speculative and non-linear methods in science communication, engagement and interdisciplinary collaboration (Horst and Michael 2011, Balmer et al. 2016, Balmer et al. 2015, Fitzgerald et al. 2014), we jointly analysed the transcript for themes, which form the basis of section headings to this paper.

5 Any account of the history of synthetic biology is likely to be both fragmentary and incomplete. Both Keller (2009) and Bensaude Vincent (2013) point to the traffic between contemporary versions of synthetic biology and earlier developments in synthetic chemistry, perhaps best epitomised by Jacques Loeb’s (1912) *The Mechanistic Conception of Life* and the work of his colleague Stéphane Leduc (1912) in *La Biologie Synthétique.* At th*e* same time, much of the initial policy and public enthusiasm for synthetic biology was premised on accounts of the field’s novelty, based in part on new techniques of gene editing.

6 Much of this coverage has focused specifically on the work of the J. Craig Venter Institute and claims about the creation of artificial life.

7 This account of synthetic biology is based on a comparison of two highly cited papers that were both published in 2005. These papers include Endy’s (2005) ‘Foundations for engineering biology’ published in *Nature* and Benner and Michael Sismour (2005) ‘Synthetic biology’ published in *Nature Reviews Genetics.* While the naming of synthetic biology as a field draws on longer history of synthetic chemistry (see for example Leduc 1912) both of these papers present alternative visions of the field whilst also being presented as constituting something of an original impulse.

8 Given the traffic between these two areas the distinction between synthetic biology and bio-nanotechnology is relatively arbitrary. In her account of the disciplinary histories of synthetic biology Bensaude Vincent (2013) cites Campos’ (2010) claim that the term ‘synthetic biology’ was coined by Carlos Bustamante at a “*Nature* cocktail party in San Francisco in 2001” (p. 18). In this sense, the naming of new fields of research is both contingent and highly charged. At the same time, Bensaude Vincent suggests that Bustamante’s approach to what would become known as synthetic biology could also be characterised as “typical of bionanotechnology” particularly given the “extensive use of Scanning Force Microscopy (SFM)” in “single-molecule manipulation and detection with optical tweezers and single-molecule fluorescence microscopy” and research on the “behaviour of biomolecular motors, molecular mechanisms of control of transcription in prokaryotes” (p. 123).

9 CRISPR-Cas9 is an acronym for Clustered Regularly Interspaced Short Palindromic Repeat and refers to “the unique organization of short, partially palindromic repeated DNA sequences found in the genomes of bacteria and other microorganisms” (Pak 2014), and has been commonly presented as a “breakthrough” gene editing technology. CRISPR-Cas9 was declared the 2015 Breakthrough of the Year by *Science*, and has been the subject of extensive public discussion – both promissory and more critical. Notably, this public commentary has also focused on questions of origin, with debate centring on the contentious ‘invention’ and ‘discovery’ of CRISPR-Cas9 techniques. For example, in recent years public commentary on CRISPR techniques has focused on alternating accounts of the ‘heroes’ of CRISPR’s development (Lander 2016) and the ‘unsung’ researchers that remain unacknowledged in these accounts (Ledford 2017b). In his review of Doudna and Sternberg’ (2017) memoir of the development of CRISPR techniques - *A Crack in Creation: Gene Editing and the Unthinkable Power to Control Evolution -* Comfort (2017) outlines the ways in which these origin narratives are fuelled by political, legal and economic strategy. In the context of competing patenting processes before the European Patent Office and the US Patent and Trademark Office Comfort (Sherkow 2018, Harrison 2018, see for example: Ledford 2017a) Comfort outlines that “after the race for discovery comes the battle for control of the discovery narrative. The stakes for the CRISPR–Cas system are extraordinarily high” (p. 30).

10 See also Nerlich (2015) and for comparison to targeting metaphors in nanomedicine see Nerlich (2015) & Bensaude-Vincent and Loeve (2014).

11 At the time of time of writing, the notion that the use of CRISPR might “cause unwanted mutations at off-target sites that resemble the on-target sequence” (Kleinstiver et al. 2016, 490) remains hotly debated. For example, a letter published in *Nature Methods* that noted that “concerns persist regarding secondary mutations in regions not targeted by the single guide RNA” and reported that “at least certain sgRNAs may target loci independently of their target in vivo” and that “the unpredictable generation of these variants is of concern” (Schaefer et al. 2017b, 547) generated significant scrutiny and was later retracted by the journal. In the retraction notice issued in *Nature Methods,* the journal editors summarised the substantial critiques of the results reported by Schaefer et al. (2017b), noting that the study “lacked key controls so that it is not possible to ascribe the observed genomic variants, with reasonable confidence, to CRISPR” (Anon 2018, 229). At the same time, the journal editors concluded by underscoring the evidentiary uncertainty regarding the possible off-target effects of CRISPR in vivo, noting that: “There is relatively little published data on genome-wide effects of in vivo CRISPR treatment. Most studies of off-target changes in CRISPR-treated organisms are not agnostic; they examine genomic sites that are algorithmically predicted to harbor off-target sequences. While this is in keeping with the known mechanism of Cas9, the enzyme could, at least in principle, have unpredicted effects on the in vivo genome.” (p. 230).

12 These claims are based on two recently reports that suggest that “genome editing by CRISPR–Cas9 induces a p53-mediated DNA damage response and cell cycle arrest in immortalized human retinal pigment epithelial cells” (Haapaniemi et al. 2018) and that “Cas9 toxicity creates an obstacle to the high-throughput use of CRISPR/Cas9 for genome engineering and screening in hPSCs. Moreover, as hPSCs can acquire P53 mutations14, cell replacement therapies using CRISPR/Cas9-enginereed hPSCs should proceed with caution, and such engineered hPSCs should be monitored for P53 function” (Ihry et al. 2018).

13 Bush Mechanics is a popular TV show, broadcast on the Australian Broadcasting Commission, which features the kinds of improvised mechanical and car maintenance practices engaged by indigenous communities in remote Australia (see: Clarsen 2002). The analogy to Bush Mechanics here seemingly works on two levels – indicating both the ‘remoteness’ of biological landscapes, and the kinds of improvisational work entailed in synthetic biology research.
